# Immunopathology of Corneal Allograft Rejection and Donor-Specific Antibodies (DSAs) as Immunological Predictors of Corneal Transplant Failure

**DOI:** 10.3390/cells13181532

**Published:** 2024-09-13

**Authors:** Natalia Olejkowska, Iwona Gorczyca, Marek Rękas, Marzena Garley

**Affiliations:** 1 Independent Researcher, 00-001 Warsaw, Poland; n.lawreniuk16@gmail.com; 2 Department of Ophthalmology, Military Institute of Medicine—National Research Institute, Szaserów 128, 04-141 Warsaw, Poland; iwnuk@wim.mil.pl (I.G.); mrekas@wim.mil.pl (M.R.); 3 Department of Immunology, Medical University of Bialystok, Waszyngtona 15A, 15-269 Bialystok, Poland

**Keywords:** keratoplasty, transplantation, cornea, human leukocyte antigens (HLAs), donor-specific antibodies (DSAs), ocular immune privilege

## Abstract

Despite tremendous developments in the field of laboratory testing in transplantation, the rules of eligibility for corneal transplantation still do not include typing of human leukocyte antigens (HLAs) in the donor and recipient or detection of donor-specific antibodies (DSAs) in the patient. The standard use of diagnostic algorithms is due to the cornea belonging to immunologically privileged tissues, which usually determines the success of transplantation of this tissue. A medical problem is posed by patients at high risk of transplant rejection, in whom the immune privilege of the eye is abolished and the risk of transplant failure increases. Critical to the success of transplantation in patients at high risk of corneal rejection may be the selection of an HLA-matched donor and recipient, and the detection of existing and/or de novo emerging DSAs in the patient. Incorporating the assessment of these parameters into routine diagnostics may contribute to establishing immune risk stratification for transplant rejection and effective personalized therapy for patients.

## 1. Introduction

Keratoplasty, or corneal transplantation, is a surgical procedure that involves replacing part or all of the thickness of the host corneal tissue with a fragment taken from a donor [1]. The history of corneal transplantation dates back to ancient times, but it was not until the 20th century that it saw rapid development. All previous attempts to transplant corneas of animal origin into humans ended in rejection of the transplanted tissue. It was not until 1905 that Eduard Konrad Zirm at the Olumnic Ophthalmic Clinic (in what is now the Czech Republic) performed the world’s first successful and documented allogeneic corneal transplantation in a patient with bilateral corneal scars after chemical burns. Unlike earlier procedures using animal tissue, Zirm used corneal tissue taken from a deceased human for keratoplasty [2,3]. Today, around the world, allogeneic corneal transplants are the most commonly performed and most successful transplants in practice. Transplants using corneal tissues from the same patient, so-called autografts, belong to sporadic procedures [4].

According to 2020 data from the Eye Bank Association of America, 66,278 eye tissues were distributed for keratoplasty, double the number of solid organ transplants (SOTs) performed in the United States. By comparison, 1269 corneal transplants and 1180 SOTs were performed in Poland during the same period [5].

Corneal transplantation is a procedure to cure many corneal conditions, making blindness from corneal causes reversible in most cases [6]. The indications for keratoplasty include pseudophakic bullous keratopathy (corneal stromal edema after cataract surgery), keratoconus, post-inflammatory scars (including scars from herpetic keratitis) scars from trauma and burns, active ulcers (bacterial, fungal, viral) unresponsive to conservative treatment, corneal degenerations and dystrophies (Fuchs dystrophy, congenital dystrophies), and retransplants. The most commonly performed procedure was penetrating keratoplasty (PK), which involves transplanting the full thickness of a corneal flap with all its layers [7,8,9,10]. Recently, lamellar transplants, in which only selected layers of the cornea are transplanted, have gained popularity due to the reduced risk of rejection [11,12,13] and better visual outcome. Pramanik et al. (2006) [14] and Thompson et al. (2003) [15] in their studies confirmed that the 10-year survival rate of corneal transplants in low-risk patients is about 90%, using only corticosteroid treatment in eye drops without systemic immunosuppression. Unfortunately, the rejection rate of corneal grafts in patients qualified for urgent corneal transplantation for infectious reasons, for secondary keratoplasty (corneal retransplantation), or with corneal neovascularization is as high as 70%, and topical and systemic use of immunosuppressive drugs proves to be insufficient [11,16,17].

Vision disorders not only prevent professional work but can lead to social isolation. Currently, corneal transplantation is the most effective therapeutic method that can restore corneal translucency and improve visual acuity in patients [10]. Discovering the sources of immune activation and neoangiogenesis in the transplanted tissue will allow for early detection of possible problems related to probable rejection of the transplanted cornea and the undertaking of appropriate therapeutic measures, increasing the chance of graft survival and thus improving the quality of life of patients [18]. HLA typing or DSA detection offers great promise for a personalized diagnostic approach, especially in patients at high risk of rejection [19].

## 2. MHC-HLA Antigens in Transplantation

**Figure 1:**
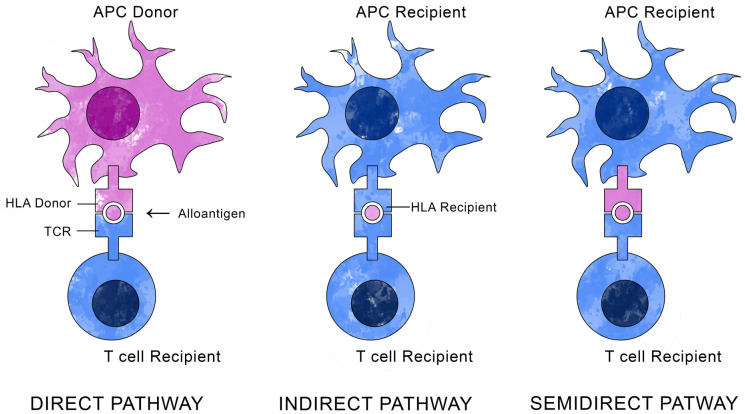
Main alloantigen recognition pathways.

Selection in terms of leukocyte antigens (HLAs, Human Leucocyte Antigens) between recipient and donor is crucial for transplant success [20]. Tissue compatibility antigens ensure the integrity of the organism and determine the biological identity of the individual. The complex of genes responsible for encoding these antigens is called the Major Histocompatibility Complex (MHC), is about 4 million base pairs long, and resides on the shorter arm of the 6th chromosome [21,22]. It has been proven that corneal transplant rejection is closely related to the MHC-HLA system, since it is the HLAs that are responsible for the recognition of own cells and foreign cells by the immune system [23,24]. Figure 1 presents a diagram of alloantigen recognition pathways. 

The figure shows the three alloantigen recognition pathways: direct, indirect, and semidirect. In the direct pathway, donor antigen-presenting cells (APCs) present alloantigen directly to recipient T cells. This reaction results in acute graft rejection. In the indirect pathway, recipient APCs present processed donor alloantigen to recipient T cells. This typical immune response results in chronic graft rejection, often with vasculopathy and antibody production. In the semidirect pathway, recipient APCs acquire donor HLA molecules, on which they present alloantigen to recipient T cells. The clinical significance of the semidirect immune response is not fully understood. The greater the match in terms of the HLA system between donor and recipient, the lower the chance of rejection occurring [25,26].

The major tissue compatibility system is highly polymorphic, so the likelihood of finding two organisms identical in terms of HLAs is negligible [27]. Expression of HLA class I antigens is present on the surface of every nucleated cell in the human body (including trophoblast cells, placenta, cornea, thymus, epithelial cells in the gastrointestinal tract and thymus, endothelial cells, fibroblasts, monocytes, keratinocytes, dendritic cells, erythroblasts, mesenchymal stem cells, and erythrocytes) [28]. In contrast, HLA class II antigens are present only on the surface of professional antigen-presenting cells (APCs), such as dendritic cells, macrophages, some endothelial cells, thymic epithelial cells, Langerhans cells (LCs), and B cells, or activated T cells [29]. HLA-matching between donor and patient plays a key role in bone marrow or kidney transplantation but is not routinely used in keratoplasty [23,30,31].

In 1969, the first monitoring studies of anti-HLA antibodies in parenchymal organ transplant patients were conducted [32,33]. Donor-Specific Antibodies (DSAs) circulating in the recipient’s serum directed against donor HLAs are of particular importance. DSAs bind to donor HLAs on the surface of the transplanted organ and activate immune mechanisms of cytotoxicity that lead to graft rejection [30,34].

## 3. Immune Privilege of the Cornea

Immune privilege of the eye is based on an evolutionary adaptation that involves the inhibition of the immune response against foreign antigens to protect sensitive structures, with preservation of visual acuity and prevention from damage caused by inflammation. There are three barriers of the immune system that make up the immune privileging of the cornea: the anatomical barrier, the cellular barrier, and the molecular barrier [35]. The first blood–eye barrier is characterized by the absence of blood and lymphatic vessels in a healthy cornea [36]. The corneal cellular barrier is associated with a small number of mature antigen-presenting cells, main among which are Langerhans cells populating the corneal epithelium [37]. It is a unique type of dendritic cells of myeloid origin, belonging to the monocyte/macrophage cell pool. The molecular barrier is composed of several components. One is the constitutive expression of Fas Cell Surface Death Receptor Ligand (FasL, or CD95L), which, by binding to the Fas receptor (Fas Cell Surface Death Receptor—CD95) present on the surface of T cells, initiates the process of apoptosis in these cells. The second component of the molecular barrier is the constitutive expression of Programmed Death Receptor 1 Ligand (PD-L1), which, by forming a complex with PD receptor-1 (CD279) on the surface of T lymphocytes, limits the activation, proliferation, and function of effector T cells. Another component of the molecular barrier consists of soluble molecules that form the immunosuppressive environment of the aqueous fluid (AqH, Aqueous Humor), key among which are pro-inflammatory cytokines, anti-inflammatory cytokines, immunoglobulins, and coagulatory and fibrinolytic proteins. The final component of this barrier is Anterior Chamber-Associated Immune Deviation (ACAID), which prevents Delayed-Type Hypersensitivity (DTH) and induces immune tolerance in the cornea [38,39,40,41,42,43,44].

Under physiological conditions, the cornea is a transparent and vascular-free tissue. Anti-angiogenic factors in the cornea are thrombospondin 1 (TSP-1), endostatin, and Pigment Epithelium-Derived Factor (PEDF) [45]. In turn, factors such as vasoactive intestinal peptide (VIP), α-melanocyte-stimulating hormone (α-MSH), and transforming growth factor β (TGF-β) show inhibitory effects on lymphatic vessel formation [46,47]. The vascular endothelial growth factor receptor 3 (VEGFR-3), which is expressed in its epithelial cells, is responsible for regulating the formation of corneal blood and lymphatic vessels, and, by binding to the proangiogenic factors VEGF-C (Vascular Endothelial Growth Factor C) and VEGF-D (Vascular Endothelial Growth Factor D), it counteracts the formation of new vessels [48]. Modulation of angiogenesis is dependent on the presence of oxygen in the cornea, as insufficient oxygen affects the activation of proangiogenic factors, which cause vascular proliferation and loss of transparency [46,49,50,51,52].

Another important element in the immune privileging of the cornea is the immunosuppressive environment that results from the presence of, among others, TGF-β, which inhibits the immune response of APCs, T cells, B cells, NK cells, and macrophages and, together with α-MSH, induces regulatory T cells (Tregs) and inhibits the production of interferon γ (INF-γ) by Helper T Cells type 1 (Th1) [53,54]. 

Transmembrane mucins—MUC1 (Mucin 1) and MUC16 (Mucin 16)—found in the corneal epithelium are also responsible for the suppressive state of the eye. MUC1 inhibits the unnecessary activation of the Toll-like receptor (TLR) and the expression of the pro-inflammatory cytokines IL-6 (Interleukin 6), IL-8 (Interleukin 8), and TNF-α (Tumor Necrosis Factor α), thereby reducing the onset of excessive inflammatory reactions in the cornea. In turn, MUC16 binds to galectin-3, a protein of the β-galactoside-binding lectin family and forms complexes on the glycocalyx surface of the ocular surface epithelium. The resulting MUC16-galectin-3 complexes inhibit the influx of extracellular molecules, including allergens, microorganisms, toxic substances, and foreign bodies, which contributes to maintaining immune homeostasis in the anterior chamber of the eye [55,56]. 

There are also factors in the cornea that affect the process of apoptosis. Examples of such molecules are the FasL, which initiates the apoptosis process of CD95 receptor T cells, and the PD-L1/B7-H1 programmed death ligand, which mediates T cell death. The process of apoptosis initiated by the Fas-FasL complex is a key mechanism for regulating the immune response. Its function is to eliminate autoreactive T lymphocytes, thus minimizing the risk of immune and autoimmune disorders in eye tissue [52,53].

In a healthy cornea, antigen-presenting cells may be in an immature state or characterized by a tolerogenic phenotype affecting weaker expression of MHC class II molecules and costimulatory molecules involved in antigen presentation. The reduced activation and response of T cells to antigens presented to them by APCs contributes to the weakened immune response in the cornea. In addition, non-classical MHC class I molecules, such as HLA-G, are present in epithelial cells, which also attenuate the immune response of effector T cells and Natural Killer Cells (NK cells) [57,58].

Another mechanism related to ocular immunotolerance has been described—the differentiation of the anterior chamber of the eye. ACAID inhibits the development of type IV hypersensitivity reactions, exhibits immunosuppressive effects against T cells and the complement system, and conditions tolerance against antigens in the anterior chamber of the eye. Such a condition ensures that no inflammatory reaction occurs inside the eyeball, which significantly affects the success of corneal transplants [59,60].

## 4. Immunopathology of Allogeneic Corneal Transplant Rejection

**Figure 2:**
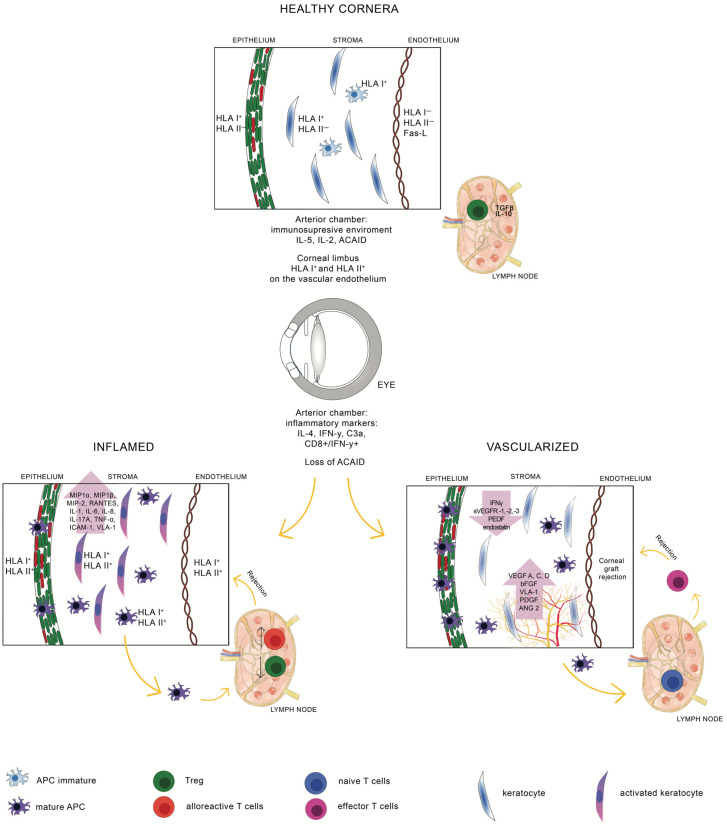
The corneal graft rejection pathways.

There are four types of corneal graft rejection, depending on the corneal layer involved. Both the most severe and the most common type of rejection of a transplanted corneal flap is endothelial rejection. This is when inflammatory cells, mainly T cells, B cells, plasma cells, dendritic cells, and macrophages, accumulate in the endothelium of the transplanted corneal flap, forming linear deposits (the so-called Khodadoust line) that damage endothelial cells. As a result of endothelial cell failure, permanent swelling of the graft and loss of its translucency manifested by decreased visual acuity can occur. Another type of graft rejection is subepithelial rejection, which, on slit-lamp examination, is characterized by the formation of subepithelial corneal infiltrates, similar to the inflammatory infiltrates characteristic of adenoviral keratitis. Rare cases of rejection include epithelial rejection, in which the immune response is directed against donor epithelial cells. The accumulation of lymphocytes in the corneal epithelium can result in epithelial failure, resulting in graft rejection. Biomicroscopy shows centripetal elevation of the corneal epithelium. The least common type of graft rejection is isolate stroma rejection, which is accompanied by neovascularization and necrosis of the corneal stroma [11,61]. Immune rejection, caused by a cellular response involving the recipient’s T cells or a humoral response involving the recipient’s antibodies directed against the donor’s leukocyte antigens exposed on the transplanted corneal cells (DSAs), is the most common cause of corneal transplant failure and usually occurs in the first year after keratoplasty. The main risk factors include neovascularization, infection (inflammation) of the eye, repeat transplantation, and younger age of the recipient. Keratoplasty in children, compared to adult patients, has a higher risk of rejection, and this is due to the impaired activity of the pediatric immune system. In inflammatory conditions, HLA class II antigens become overexpressed and uncontrolled on epithelial cells, stromal cells (keratinocytes), and endothelial cells, which in turn leads to excessive and abnormal stimulation of lymphocytes, mainly type 1 helper lymphocytes (Th1) [62]. The result of increased inflammation is damage to corneal tissue. In the transplanted cornea, during the process of neovascularization, not only new blood vessels grow but also lymphatic vessels [63]. The new lymphatic vessels create a so-called afferent pathway of graft rejection for the recipient’s mature antigen-presenting cells (APCs), which transport donor antigens from the transplanted corneal tissue to nearby lymph nodes. In the lymph nodes, naive T cells (Th0) are activated, leading to clonal expansion of Th1 lymphocytes that secrete pro-inflammatory cytokines (e.g., IFN-γ, IL-2) [64]. The cascade of these reactions contributes to an increased immune response to the graft, which can eventually lead to corneal graft rejection [33,65]. Macrophages, NK cells, and granulocytes have also been shown to accumulate at the site of graft rejection. However, the specific interactions between the aforementioned types of immune cells and corneal tissue at the site of rejection have not yet been studied in detail [66]. Figure 2 shows the corneal graft rejection pathways.

The figure shows two possible pathways for corneal graft rejection. In healthy corneas, HLA I antigens are detected on the epithelium and keratocytes. HLA I and HLA II antigens present on the limbal endothelium are not detected [67]. There are no APCs or other inflammatory cells, only a small number of immature APCs present in the stroma [39]. In the anterior chamber, the immunosuppressive environment is provided by ACAID; the protective effect of IL-2 and IL-5 [68]; the presence of FasL on corneal epithelial and endothelial cells ensuring apoptosis of Fas+ cells [69]; and drainage of the eye in the presence of IL-10 and TGFβ secreted by TregFoxp3+ in lymph nodes [70]. In inflammation, HLA I and II antigens are induced on the endothelium, expressed on the epithelium, keratocytes, and endothelium, with a large number of activated keratinocytes and mature APCs [71,72]. The balance between anti-inflammatory and pro-inflammatory factors is disturbed, and IL-4, IFN-γ, and C3a are present. An increase in the expression of pro-inflammatory cytokines (IL-1, IL-6, IL-8, IL-17A, TNF-α), chemokines (MIP-1α, MIP-1β), RANTES, and adhesion molecules (ICAM-1, VLA-1) is observed [68,73,74,75]. Stimulated mature APCs present donor antigens to naive T cells in lymph nodes. Effector T lymphocytes after clonal expansion produce IL-2, IFN-γ, and TNF-α, leading to endothelial cell apoptosis, resulting in corneal edema and loss of graft transparency. In a vascularized corneal graft, proinflammatory mediators stimulate neovascularization of blood and lymphatic vessels, which facilitates the transfer of donor antigens by mature APCs to lymph nodes and presentation to naive T cells (Th0). Clonal expansion of Th1 cells leads to graft rejection [5].

Current research is focused on finding early predictors of graft rejection. Flynn et al. examined the composition of the aqueous fluid (AqH) in patients after transplant rejection. The researchers observed an increased percentage of CD14+ leukocyte population, thus confirming the role of APCs in rejection, as well as significantly elevated levels of IL-6, the chemokines CXCL10 (C-X-C Motif Chemokine Ligand 10), CCL2 (C-C Motif Chemokine Ligand 2), and CCL3 (C-C Motif Chemokine Ligand 3), and eotaxin. CXCL10, CCL2, CCL3, and eotaxins are involved in the recruitment of various types of immune cells, including macrophages and lymphocytes, to the site of transplant rejection. Controlling chemokine levels may be one of the potential therapeutic targets in preventing transplant rejection. Importantly, in corneal transplant patients, aqueous fluid aspiration did not cause complications, making it an important and useful diagnostic material in the future [76].

Other researchers in a mouse model of corneal transplantation have shown that neutralization of vascular endothelial growth factor-A (VEGF-A) results in inhibition of angiogenesis, lymphangiogenesis, and macrophage recruitment to the transplanted corneal tissue [77]. Current human clinical trials for the treatment of ocular diseases, including corneal neovascularization, are based on the use of the approved drug bevacizumab, which is a monoclonal antibody that binds anti-VEGF-A. Topical application of this drug in humans effectively inhibits the excessive proliferation of blood and lymphatic vessels in the cornea [77,78,79]. Recent studies by January et al. (2024) provide encouraging data on DSA and AMR inhibition with tocilizumab, an IL-6 inhibitor, in lung transplant patients. Tocilizumab resulted in DSA reduction, less DSA growth, a lower incidence of new DSAs, and lower graft failure rates [80].

Intensive research is underway into the potential use of regulatory T cells in transplantation and in the treatment of autoimmune diseases. Currently in clinical trials, the most promising strategy to increase the number of regulatory T cells is the so-called adoptive transfer of Treg cells multiplied ex vivo. In their study, Chauhan et al. found that Treg cells isolated from blood, when multiplied, activated and intravenously administered to mice after corneal transplantation, contributed to a significant improvement in transplant survival. Allospecific Treg cells from transfer were characterized by a stronger immunosuppressive response in contrast to naive Treg cells [81]. Another therapeutic option is to increase the immunosuppressive potential of regulatory T cells by capturing IL-2 (Interleukin 2) from the response microenvironment. This has the effect of reducing the availability of this cytokine to other T cell subpopulations, resulting in an inhibition of effector cell activity through increased expression of the transcription factor FoxP3 (Forkhead Box P3) and increased production of the immunosuppressive molecules IL-10 and TGF-β [82].

The proteins characteristic of Treg lymphocytes include CTLA-4 (cytotoxic T lymphocyte antigen 4). By binding to the costimulatory molecules CD80/CD86 found on the surface of antigen-presenting cells, CTLA-4 inhibits the costimulatory signal necessary for full activation of the T cells. A study by Hoffmann et al. demonstrated improved corneal graft survival with systemic administration of CTLA-4-Ig fusion protein. In turn, Kagaya et al. showed that blocking antigen presentation with anti-CD80/CD86 antibodies significantly reduces the rejection rate of transplanted ocular tissue [83,84,85].

## 5. Role of Antibodies in Corneal Transplant Rejection

After corneal transplantation, donor HLAs appear in the recipient’s body, which the patient’s immune system can recognize as foreign antigens and induce an immune response against these antigens. The recipient’s foreign antigen-activated B cells can differentiate into plasma cells and produce antibodies specific for donor leukocyte antigens (DSAs). DSAs circulating in the recipient’s serum, after binding to specific donor HLAs on the cell surface of the transplanted tissue, activate cytotoxicity mechanisms in a series of immune reactions [5].

There are studies supporting the possible role of donor HLA-specific antibodies (DSAs) in corneal transplant rejection. Roy et al. (1992) demonstrated the negative effect of post-transplant DSAs produced (both in recipients compatible or incompatible in HLA locus A and B with the donor), which increase the risk of endothelial rejection [86]. Hahn et al. (1995) concluded from their study that lymphocytotoxic antibodies, particularly those directed against the donor’s HLA class I, in patients after keratoplasty, affect immune-mediated graft failure [87]. However, other researchers have found that the presence of DSAs has no predictive value in corneal graft rejection. For example, Jager et al. (1994) argued that a group of patients with keratoconus and after graft rejection due to non-immune causes (e.g., caused by corneal decompensation) had a significantly higher incidence of DSAs than healthy controls [88]. In contrast, Hargrave et al. (2003) found that transplanted corneal tissue stimulates the production of allospecific antibodies directed against tissue compatibility antigens, while the presence of IgG alloantibodies alone does not correlate with corneal graft rejection and can occur even in the absence of DSAs [89].

In 2012, Sel et al. divided their study group into 45 low- and high-risk corneal transplant rejection recipients and observed these patients for 18 months for the presence of DSAs before and after transplantation. The authors observed that 75% of patients with preformed DSAs suffered from immune complications, including four cases that resulted in complete graft loss within the first two months. In contrast, 77% of recipients without preformed DSAs had no immune complications throughout the follow-up [90].

As early as 1969, Patel and Terasaki discovered the negative effect of antibodies directed against MHC antigens in organ transplants [33]. Major risk factors for the production of anti-HLA antibodies include blood transfusions, pregnancies, and a history of transplantation. It is now known that not only the cellular response but also the humoral response plays a key role in graft loss. Some DSAs use complement binding capacity as a major mechanism of solid organ transplant failure (SOT). In addition, studies have shown that the presence of DSAs that bind the C1q component is an independent factor in antibody-mediated rejection (AMR) in renal transplant rejection [33,91,92,93,94].

The adaptive immune response, otherwise known as the acquired response, is antigen-specific, but its role in corneal transplant rejection has not yet been thoroughly described. Goslings et al. (1999), in their study on mice suffering from immune deficiencies—B-lymphocyte deficiency and complement component deficiency, showed that rejection can occur even in the absence of complement-component-binding antibodies [95]. Four years later, Hargrave et al. showed that endothelial cells not only play a major role in maintaining corneal transparency but are also the most susceptible to complement-dependent or -independent lysis by cytotoxic antibodies against transplant donor antigens, i.e., DSAs [89].

Allospecific antibodies are not always the cause of corneal graft decompensation, while they can negatively affect graft maintenance in a complement-dependent or -independent manner. Depending on the type of corneal graft rejection, a different treatment strategy should be used [5].

The current diagnostic challenge is to clearly distinguish potentially benign DSAs from harmful DSAs. The DSA concentration and binding strength to the antigen must be assessed. Hug et al. developed flow-induced dispersion analysis (FIDA), which provides a measure of DSA affinity in patient serum samples along with DSA concentration, which has important clinical value [96].

## 6. Conclusions

The knowledge collected over many years about the corneal microenvironment and the importance of its immune privilege is vast but still needs to be deepened. Unfortunately, most studies are based on mouse models, so there is a need for more studies on patients to better understand the mechanisms of rejection and tolerance of corneal allografts. Currently, DSA monitoring has become the cornerstone of AMR risk stratification in organ transplant recipients, but it is not a routinely used procedure in keratoplasty. Establishing a link between pre-existing and/or de novo formed DSAs and the corneal rejection process in patients requires more randomized clinical trials. It is important to remember that, unlike in SOTs, we distinguish between four forms of graft rejection in corneal transplant patients, which can overlap. It seems reasonable to assess the immune risk of rejection in corneal recipients with DSAs before and after transplantation. In these patients, DSAs may impair the therapeutic effect and accelerate rejection of the transplanted ocular tissue. Special attention should be paid to recipients at high risk of rejection, in whom diagnostic management based on assessment of HLA compatibility with the donor and monitoring of donor-dependent antibodies appears to be crucial for transplant success. Proper diagnosis in this group of patients can reduce the need for retransplantation and reduce the risk of complications after rejection of an incompatible graft.
